# The Trials of a Dyspeptic

**Published:** 1877-04

**Authors:** 


### THE TRIALS OF A DYSPEPTIC.

Well, it *is* a trial to be a dyspeptic—a constant trial. You are sick if you eat, and eat if you are sick, and for the life of you, you cannot tell under which formula you are best conditioned. Let your diet be strictly after the graham biscuit and mush order, (stuff no more digestible than so much sand, we desire to observe), and your head, in company with your stomach, will ache like fury all next day. Partake of terrapin soup and champagne for supper, to appease your famishing appetite, and quite as likely as not you will feel like a fighting-cock for a week to come, only to be thrown into a week's headache at the very next attempt of that sort. So you are perplexed and annoyed to determine when and what to eat. When a fellow is quite as much sick when he "takes care of himself," i. e., lives upon starvtion diet, partaking of oat meal, beef tea and similar slops, as when he goes in for a real hearty dinner of roast beef and its accessories, what in fury is he to do? It is a trite saying but somewhat applicable to this case, "you might as well die for a sheep as a lamb," and surely, why not?

The trials of the dyspeptic are sometimes funny as well as distressing. It is amusing to see him sit in his easy chair, after his moderate supper, feel his pulse, and, every time his heart gives a skip his eyes give a blink, his thoughts a summersault, and his tongue an expression—which becomes intelligible in "heart disease." Every time that heart skips, he does likewise, until he brings up in his physician's office and at once demands an examination of that mysterious organ. He tells the doctor he *knows* he has "heart disease," and if he has, he don't want to know it. When the physician declares it to be only a functional disturbance, the result of indigestion, he does not believe a word of it, but goes straightway home, makes his will, gives a few parting instructions to his wife, then goes to bed, but sits bolt upright, with fingers upon his pulse, calmly awaiting his end. But it never comes. Then he resorts to digitalis—takes from ten to fifteen drops twice a day, until his heart becomes so slow and confoundedly regular as to be monotonous, not to say annoying. He therefore concludes to hurry it up a trifle, with a stimulant, 'cause he don't want it to stop entirely, you know. Takes brandy —tastes good—touches the spot—has some more, and wakes up in the morning with a pain in his stomach and a worse one in his head. Suddenly, a pain under his shoulder manifests itself. That smacks of consumption, and forthwith he has his lungs examined—cod-liver oil three times daily. In a day or two, a pain is found in the small of his back—that hints at Bright's disease, and argues loudly for a microscopic examination of the secretions—citrate of potash and lemon juice. Scarcely have these pains been analyzed and classified ere an ache has been developed under the short ribs—"liver complaint" is at once suggested, and pellets of podophyllin indulged in nightly. At last, this poor dyspeptic comes to the inevitable—a tape-worn, and that is the sum of all misery. To have three or four hundred feet of that jointed, squirming creature fastened onto and into the mucous membrane of his entire alimentary canal, is a condition not to be tolerated for an instant, therefore, pumpkin seed tea and castor oil. As the remedies increase, so do the "symptoms," until it becomes far easier to enumerate the aches and pains that are not apparent than those that are.

These be a few of the trials and perplexities that beset the ways of the dyspeptic. They are mournful, yet comical, but always render the possessor entitled to the sympathy and indulgence of his friends. The dyspeptic is an unmitigated nuisance in society—but never tell him so, poor fellow, he is about as miserable as he can be. While you enjoy your hearty meal, he sits by and nibbles at a graham cracker or sips an oat meal porridge. While you are constructing rings from your fragrant Havana, he bolts for fresh air and some digester for his frugal meal. Well, what of it? We have only to say that, if a vast majority of dyspeptics would cease from drinking slops and chewing dry, hard, unnourishable crackers and the like, and eat three wholesome but moderate meals a day, consisting of rare roast beef, eggs, milk, and sparingly of vegetables, they would be much better off and have fewer reasons for complaint. It is our deliberate opinion, that a stomach that can digest oatmeal, cracked wheat, graham mush, *et hoc genus omne*, can far easier dispose of the more nutritious foods mentioned, because of their being infinitely easier of digestion and assimilation. In a word, cease making your stomach a receptacle for slops, and begin the eating of wholesome food, at seasonable hours and in moderate quantities.

